# Nonclassical MHC Ib-restricted CD8^+^ T Cells Recognize *Mycobacterium tuberculosis*-Derived Protein Antigens and Contribute to Protection Against Infection

**DOI:** 10.1371/journal.ppat.1005688

**Published:** 2016-06-07

**Authors:** Shaobin Shang, Sarah Siddiqui, Yao Bian, Jie Zhao, Chyung-Ru Wang

**Affiliations:** Department of Microbiology and Immunology, Feinberg School of Medicine, Northwestern University, Chicago, Illinois, United States of America; Portland VA Medical Center, Oregon Health and Science University, UNITED STATES

## Abstract

MHC Ib-restricted CD8^+^ T cells have been implicated in host defense against *Mycobacterium tuberculosis* (Mtb) infection. However, the relative contribution of various MHC Ib-restricted T cell populations to anti-mycobacterial immunity remains elusive. In this study, we used mice that lack MHC Ia (K^b-/-^D^b-/-^), MHC Ia/H2-M3 (K^b-/-^D^b-/-^M3^-/-^), or β_2_m (β_2_m^-/-^) to study the role of M3-restricted and other MHC Ib-restricted T cells in immunity against Mtb. Unlike their dominant role in *Listeria* infection, we found that M3-restricted CD8^+^ T cells only represented a small proportion of the CD8^+^ T cells responding to Mtb infection. Non-M3, MHC Ib-restricted CD8^+^ T cells expanded preferentially in the lungs of Mtb-infected K^b-/-^D^b-/-^M3^-/-^ mice, exhibited polyfunctional capacities and conferred protection against Mtb. These MHC Ib-restricted CD8^+^ T cells recognized several Mtb-derived protein antigens at a higher frequency than MHC Ia-restricted CD8^+^ T cells. The presentation of Mtb antigens to MHC Ib-restricted CD8^+^ T cells was mostly β_2_m-dependent but TAP-independent. Interestingly, a large proportion of Mtb-specific MHC Ib-restricted CD8^+^ T cells in K^b-/-^D^b-/-^M3^-/-^ mice were Qa-2-restricted while no considerable numbers of MR1 or CD1-restricted Mtb-specific CD8^+^ T cells were detected. Our findings indicate that nonclassical CD8^+^ T cells other than the known M3, CD1, and MR1-restricted CD8^+^ T cells contribute to host immune responses against Mtb infection. Targeting these MHC Ib-restricted CD8^+^ T cells would facilitate the design of better Mtb vaccines with broader coverage across MHC haplotypes due to the limited polymorphism of MHC class Ib molecules.

## Author Summary

Tuberculosis, the disease caused by *Mycobacterium tuberculosis* (Mtb), remains a major global health burden. As T cells are crucial to the control of Mtb infection, it is imperative to decipher the role of different T cell subsets in anti-Mtb immunity for the development of more effective vaccines. While the contribution of conventional T cells to protective immunity against Mtb is well established, the involvement of unconventional T cells is less clear. In this study, we used mutant mice that lack distinct MHC I molecules to characterize immune responses mediated by unconventional T cells during Mtb infection. We found that unconventional CD8^+^ T cells preferentially expanded in the lung after Mtb infection. These CD8^+^ T cells responded to numerous mycobacterial antigens, produced multiple cytokines, and contributed to protection against Mtb. A large proportion of unconventional T cells induced by Mtb infection are Qa-2 restricted CD8^+^ T cells, suggesting this group of T cells may play a greater role in anti-mycobacterial immunity than other unconventional T cell populations that have been characterized previously. Targeting these unconventional T cells may facilitate the design of TB vaccines that are universally effective in ethnically diverse populations due to the non-polymorphic nature of MHC Ib molecules.

## Introduction

Tuberculosis (TB), an infectious disease caused by *Mycobacterium tuberculosis* (Mtb), remains one of the world’s deadliest communicable diseases, with 1.5 million deaths annually [1]. Due to the emergence of multidrug-resistant Mtb strains, co-infection with HIV, and the failure of BCG vaccine to control adult pulmonary TB [1, 2], there is an urgent need for new and more effective TB vaccines. However, achieving this goal relies on further investigation of the properties of protective T cells during Mtb infection [3]. It is well established that immune protection against Mtb infection is dependent on a robust Th1 response, mediated by CD4^+^ T cells [4–7], while CD8^+^ T cells are required for optimal immunity [8–11]. The cytokines IL-12, IFN-γ and TNF-α are critical for the control of Mtb infection [12]. Current subunit vaccine candidates target conventional CD4^+^ and MHC Ia-restricted CD8^+^ T cells [13]. However, increasing evidence shows that unconventional T cells restricted by MHC Ib molecules can recognize distinct types of microbial antigens and may contribute to host defense against microbial infection [14, 15]. Yet, it remains unclear whether MHC Ib-restricted CD8^+^ T cells play a protective role during Mtb infection and which MHC Ib molecules may be involved in anti-mycobacterial immunity.

MHC Ib molecules are structurally similar to MHC Ia molecules and associated with β_2_-microglobulin (β_2_m) [14]. Unlike MHC Ia molecules, MHC Ib molecules exhibit limited polymorphism, making them attractive targets for vaccine development [14, 15]. The mammalian genome encodes many MHC Ib molecules though only a few are known to have immunological function. These include H2-M3 (M3), Qa-1/HLA-E, Qa-2/HLA-G, CD1 and MHC-related gene 1 (MR1) in mice and/or humans [14]. T cells restricted by MHC Ib molecules have been implicated in host defense against Mtb in humans and mice [15]. In particular, M3-restricted CD8^+^ T cells recognize several *N*-formylated peptides derived from Mtb [16] and vaccination of mice with dendritic cells pulsed with *N*-formylated Mtb peptides conferred protection against Mtb in mice [17]. CD1d-restricted iNKT cells, which recognize self and/or microbial lipids [18, 19], can be activated by Mtb-infected macrophages and lead to the control of intracellular mycobacteria through the production of GM-CSF [20, 21]. In addition, Mtb lipid-specific group 1 CD1-restricted T cells were detected in patients with active or latent TB infection [22, 23] and participated in host adaptive immune responses to Mtb in human group 1 CD1 transgenic mice [24, 25]. Recently, MR1-restricted mucosal-associated invariant T cells (MAIT), which recognize vitamin B metabolites during bacterial infection [26], were also shown to contribute to anti-mycobacterial immunity [27, 28]. In addition, HLA-E, the human homolog of mouse Qa-1, presents Mtb-derived peptides to cytotoxic CD8^+^ T cells [29, 30]. Although these MHC Ib-restricted T cells were detected or induced following immunization or infection, their relative contribution during Mtb infection has yet to be defined. Furthermore, it is unclear whether other MHC Ib molecules are involved in antigen presentation to T cells during Mtb infection.

Previous studies have shown that MHC Ia-deficient mice were more resistant to Mtb infection than β_2_m-deficient mice [31, 32]. However, it is unclear whether MHC Ib-restricted CD8^+^ T cells contribute to the observed protection in these studies because β_2_m^-/-^ mice have aberrant iron metabolism and impaired innate immunity aside from lacking both MHC Ia and Ib-restricted CD8^+^ T cells [33]. In addition, it is unclear whether M3 plays a dominant role in MHC Ib-mediated immune responses against Mtb infection, as was the case in *Listeria* infection [34]. In this study, we used mice that lack MHC Ia (K^b-/-^D^b-/-^), MHC Ia/M3 (K^b-/-^D^b-/-^M3^-/-^) [34] or β_2_m (β_2_m^-/-^) to study the role of M3-restricted and other MHC Ib-restricted CD8^+^ T cells in immunity against Mtb aerosol infection. We found Mtb-infected K^b-/-^D^b-/-^M3^-/-^ mice do not have a significantly reduced number of CD8^+^ T cells as compared to K^b-/-^D^b-/-^ mice, suggesting that MHC Ib molecules other than M3 are responsible for the development of a robust CD8^+^ T cell response to Mtb in the absence of MHC Ia molecules. These non-M3, MHC Ib-restricted CD8^+^ T cells recognized Mtb-derived protein antigens, expanded preferentially in the lungs of K^b-/-^D^b-/-^M3^-/-^ mice and contributed to protective immunity against Mtb. Furthermore, a large proportion of these expanded CD8^+^ T cells were restricted to MHC Ib molecule Qa-2, suggesting that this is a new T cell population that participate in immune responses against Mtb infection.

## Results

### Non-M3, MHC Ib-restricted CD8^+^ T cells contribute to protective immunity against Mtb

**Fig 1:**
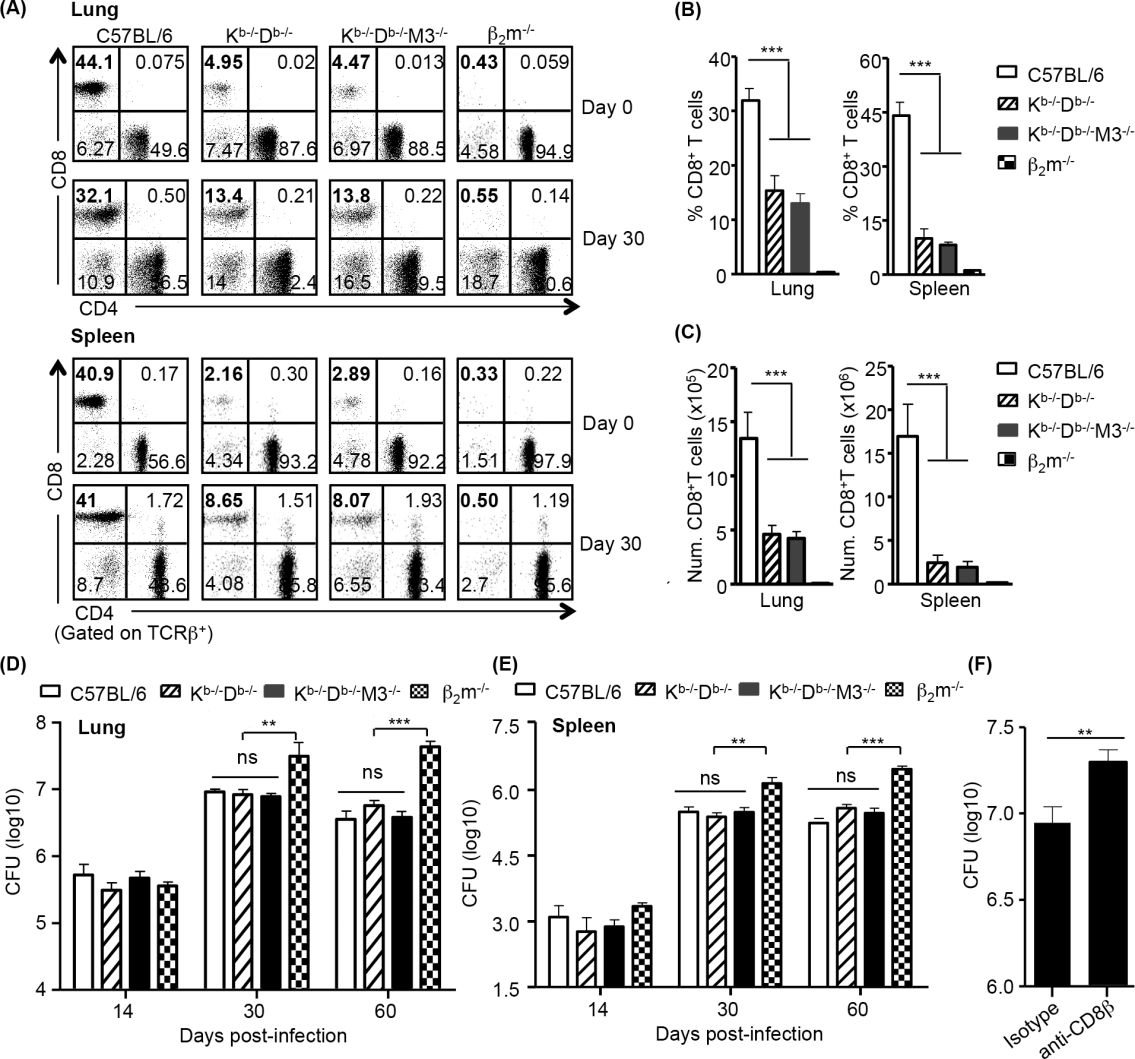
Non-M3, MHC Ib-restricted CD8^+^ T cells contribute to protective immunity against Mtb. C57BL/6, K^b-/-^D^b-/-^, K^b-/-^D^b-/-^M3^-/-^ and β_2_m^-/-^ mice were infected with aerosolic Mtb H37Rv (~200 CFU) and sacrificed at indicated time-points. (A) Representative dot-plots depict CD8^+^ and CD4^+^ T cell populations from indicated mice before infection or at day 30 post-infection. Numbers indicate the percentage of cells in each quadrant in the TCRβ^+^ population. (B, C) Bar graphs depict the mean ± SEM of the percentage (B) and total number (C) of CD8^+^ T cells in the lung and spleen of indicated mice at day 30 post-infection. Data shown are from one of three independent experiments with 3 mice in each group. (D, E) Comparison of CFU between C57BL/6 (n = 5–8), K^b-/-^D^b-/-^ (n = 9–14), K^b-/-^D^b-/-^M3^-/-^ (n = 7–15) and β_2_m^-/-^ (n = 4–6) mice in the lung (D) and spleen (E) at day 14, 30 and 60 post-infection. Data shown are pooled from three independent experiments. (F) Bacterial burden in the lung of K^b-/-^D^b-/-^M3^-/-^ mice received CD8 depleting mAb or control IgG at day 28 post-infection. Data shown are the mean ± SEM from 9 mice per group. *P <0.05, **P <0.01, ***P <0.001, ns, no statistical significance.

We have previously demonstrated that M3-restricted CD8^+^ T cells expanded extensively during *Listeria* infection and played a prominent role in host defense against *Listeria* [34]. To test whether M3-restricted CD8^+^ T cells play similar roles during Mtb aerosol infection, we first compared CD8^+^ T cell responses in the lungs and spleens of Mtb-infected C57BL/6 (B6), K^b-/-^D^b-/-^, K^b-/-^D^b-/-^M3^-/-^ and β_2_m^-/-^ mice. Consistent with previous reports, almost no CD8^+^ T cells were detected in Mtb-infected β_2_m^-/-^ mice. Interestingly, we found that CD8^+^ T cells in the lungs and spleens of K^b-/-^D^b-/-^M3^-/-^ and K^b-/-^D^b-/-^ mice expanded to a similar extent following Mtb infection, although the total numbers of CD8^+^ T cells in both mouse strains remained lower than those in B6 mice (Fig 1A–1C). Furthermore, Mtb antigen-specific CD8^+^ T cell responses in K^b-/-^D^b-/-^ mice were not reduced upon stimulation with K^b-/-^D^b-/-^M3^-/-^ BMDCs compared to stimulation with K^b-/-^D^b-/-^ (M3-sufficient) BMDCs (S1 Fig). These data suggest that among the β_2_m-dependent CD8^+^ T cells, H2-M3-restricted CD8^+^ T cells only represent a small proportion of the CD8^+^ T cells responding to Mtb infection and that other MHC Ib-restricted CD8^+^ T cells expand significantly during infection.

To evaluate the protective capacity of non-M3 MHC Ib-restricted CD8^+^ T cells during Mtb infection, we compared the bacterial burden in both the lungs and spleens of infected B6, K^b-/-^D^b-/-^and K^b-/-^D^b-/-^M3^-/-^ mice with that of infected β_2_m^-/-^ mice. Mtb-infected K^b-/-^D^b-/-^ and K^b-/-^D^b-/-^M3^-/-^ mice showed no differences in bacterial burden in the lungs and spleens over the course of infection, supporting the notion that M3-restricted T cells do not play a prominent role in anti-mycobacterial immunity. However, K^b-/-^D^b-/-^M3^-/-^ mice had significantly lower bacterial loads than β_2_m^-/-^ mice at day 30 and day 60 post-infection (Fig 1D and 1E), suggesting that non-M3, MHC Ib-restricted CD8^+^ T cells play a protective role during Mtb infection. Moreover, K^b-/-^D^b-/-^M3^-/-^ and B6 mice had comparable bacterial burden in the lungs and spleens over the course of infection (Fig 1D and 1E), suggesting that the presence of MHC-Ib restricted T cells can compensate for the lack of MHC-Ia-restricted CD8^+^ T cells to control Mtb infection.

To determine whether non-M3, MHC Ib-restricted CD8^+^ T cells mediated the protective effect observed in Mtb-infected K^b-/-^D^b-/-^M3^-/-^ mice, Mtb-infected K^b-/-^D^b-/-^M3^-/-^ mice were repeatedly treated with anti-CD8β mAb or control Ab and the bacterial burden in the lungs of these two groups of mice were compared at day 30 post-infection. As shown in Fig 1F, depletion of CD8^+^ T cells in Mtb-infected K^b-/-^D^b-/-^M3^-/-^ mice resulted in a significant increase in bacterial burden in the lungs compared to mice that received control rat IgG antibody. These results demonstrated that non-M3, MHC Ib-restricted CD8^+^ T cells contribute to the protective immunity against Mtb.

### Non-M3, MHC Ib-restricted CD8^+^ T cells preferentially expand in the lung and express elevated levels of KLRG1 during Mtb infection

**Fig 2:**
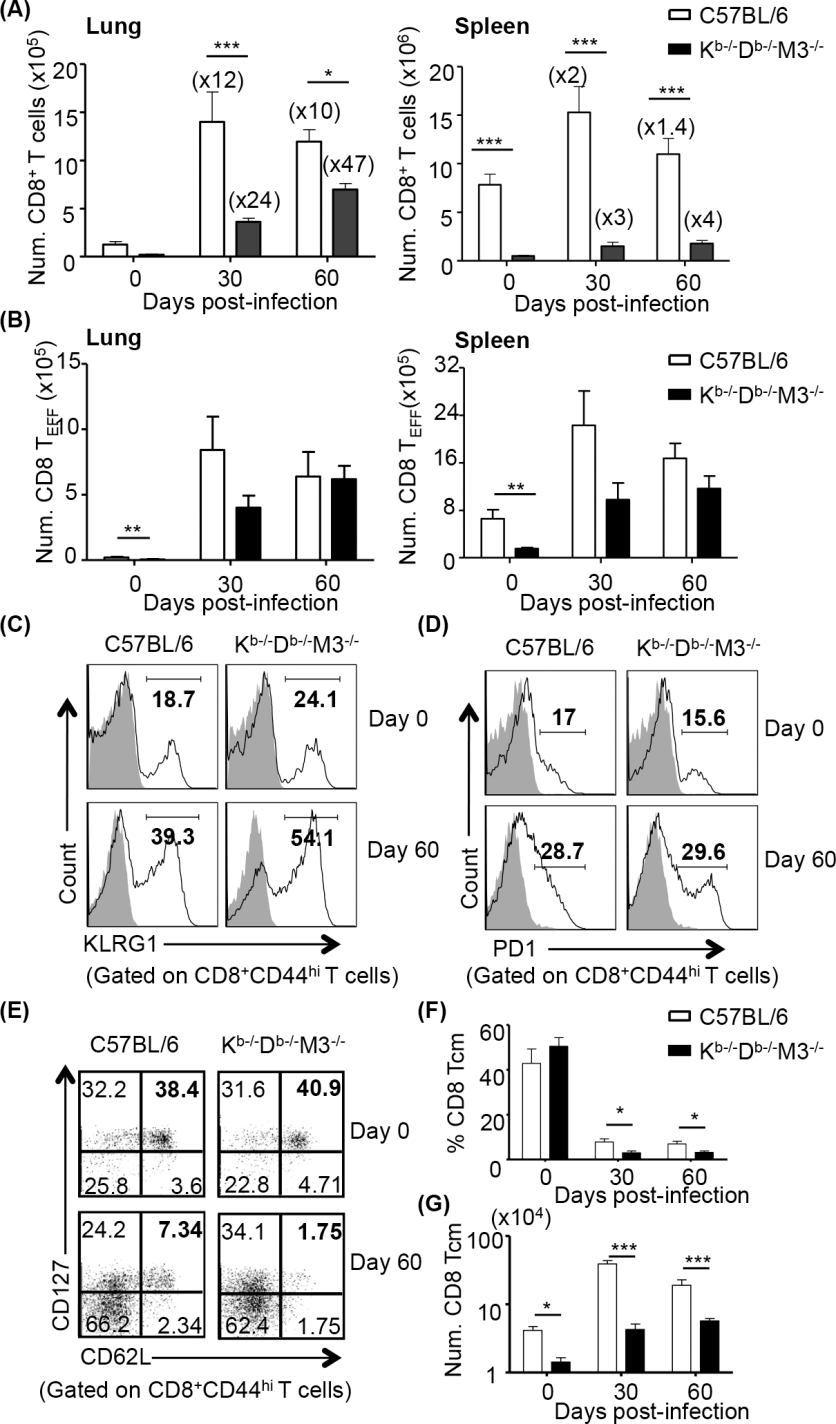
Non-M3, MHC Ib-restricted CD8^+^ T cells preferentially expand in the lung and express elevated levels of KLRG1 during Mtb infection. WT and K^b-/-^D^b-/-^M3^-/-^ mice were sacrificed at indicated time-points, single cells from the lung and spleen were prepared for phenotypic analysis of CD8^+^ T cells by flow cytometry. (A) Kinetic changes of total number of CD8^+^ T cells in the lung and spleen from C57BL/6 (n = 4–9) and K^b-/-^D^b-/-^M3^-/-^ (n = 7–9) mice after infection. Numbers in bracket indicate fold changes of expansion at indicated time points after infection. (B) Kinetic changes of total number of CD44^hi^CD62L^lo^CD8^+^ effector T cells (T_EFF_) in lung and spleen from C57BL/6 (n = 4–6) and K^b-/-^D^b-/-^M3^-/-^ (n = 4–7) mice during infection. (C, D) Representative histograms show the expression of KLRG1 (C) and PD-1 (D) on CD8 T_EFF_ cells from the lung at day 0 and day 60 post-infection. Grey solid areas indicate isotype control. Data shown are representative of three independent experiments. (E) Representative dot plots depict CD127 expression on CD8^+^ effector and memory (CD44^hi^CD62L^hi^) cells in the lungs of indicated mice before infection or at day 60 post-infection. (F, G) Bar graphs depict the mean ± SEM of the percentage (F) and total number (G) of CD8^+^ Tcm. (CD127^+^CD44^hi^CD62L^hi^) cells in the lung of C57BL/6 (n = 4–6) and K^b-/-^D^b-/-^M3^-/-^ (n = 4–7) at indicated time points after infection. *P <0.05, **P <0.01, ***P <0.001.

Since there were no significant differences in the total number of CD8^+^ T cells and bacterial loads between K^b-/-^D^b-/-^ and K^b-/-^D^b-/-^M3^-/-^ mice after Mtb infection, we used K^b-/-^D^b-/-^M3^-/-^ mice in following experiments to further characterize non-M3, MHC Ib-restricted CD8^+^ T cell responses during Mtb infection. Kinetic analysis of CD8^+^ T cells in different organs showed that CD8^+^ T cells from K^b-/-^D^b-/-^M3^-/-^ mice expanded almost 50 fold in the lung but only 3–4 fold in the spleen by day 60 post-infection (Fig 2A), suggesting that non-M3, MHC Ib-restricted CD8^+^ T cells expand more in the lung than in the spleen. Differential expansion of CD8^+^ T cells in the lung was also observed in Mtb-infected B6 mice (mostly MHC Ia-restricted), but the degree of expansion (10–12 fold) was less vigorous than that in K^b-/-^D^b-/-^M3^-/-^ mice (Fig 2A). In addition, the absolute numbers of CD8^+^ T cells with CD44^hi^CD62L^lo^ effector phenotype (CD8^+^ T_EFF_) were comparable between Mtb-infected K^b-/-^D^b-/-^M3^-/-^ and B6 mice, even though in naïve animals, K^b-/-^D^b-/-^M3^-/-^ mice had significantly fewer CD8^+^ T_EFF_ cells than B6 mice (Fig 2B). These results suggest that Mtb infection induces a more robust expansion CD8^+^ T_EFF_ cells in K^b-/-^D^b-/-^M3^-/-^ mice than CD8^+^ Teff cells in wildtype mice.

Conventional effector CD8^+^ T cells were shown to gradually lose function or become exhausted in the chronic LCMV infection model [35]. The elevated expression of several cell surface receptors including programmed death-1 (PD-1) and killer cell lectin-like receptor G1 (KLRG1) has been correlated with functional exhaustion and terminal differentiation of effector CD8^+^ T cells [35]. Most of the MHC class Ib-restricted CD8^+^ T cells in naïve K^b-/-^D^b-/-^ or K^b-/-^D^b-/-^M3^-/-^ mice exhibit an activated T cell phenotype (CD44^hi^, CD11a^hi^, and CD122^+^) [31, 34]; however, it is not clear whether MHC Ib-restricted CD8^+^ T cells would develop a similar phenotype during chronic Mtb infection. We found that at the chronic infection stage at day 60 post-infection, both K^b-/-^D^b-/-^M3^-/-^ and B6 mice had increased expression of KLRG-1 and PD-1 on CD8^+^ T_EFF_ cells compared to naïve controls. In addition, CD8^+^ T_EFF_ in K^b-/-^D^b-/-^M3^-/-^ mice expressed a higher level of KLRG1 than CD8^+^ T_EFF_ in B6 mice, whereas no difference in PD-1 expression on CD8^+^ T_EFF_ cells was detected between K^b-/-^D^b-/-^M3^-/-^ and B6 mice (Fig 2C and 2D). To further address the question whether activated CD8^+^ T cells in Mtb-infected K^b-/-^D^b-/-^M3^-/-^ mice can develop into long-lived memory precursor cells, we compared the expression of CD127, the IL-7 receptor α, on CD44^hi^CD8^+^ T cells in Mtb-infected K^b-/-^D^b-/-^M3^-/-^ and B6 mice. We found a significantly lower proportion of memory (CD44^hi^CD62L^hi)^ CD8^+^ T cells in Mtb-infected K^b-/-^D^b-/-^M3^-/-^ mice expressed CD127 as compared to those in B6 mice (Fig 2E–2G). These results suggest that MHC Ib-restricted CD8^+^ T cells acquire a terminally differentiated phenotype during Mtb infection and most of these CD8^+^ T cells are short-lived effectors.

### Mtb antigen stimulation induces high frequency of IFN-γ producing CD8^+^ T cells in Mtb-infected K^b-/-^D^b-/-^M3^-/-^ mice

**Fig 3:**
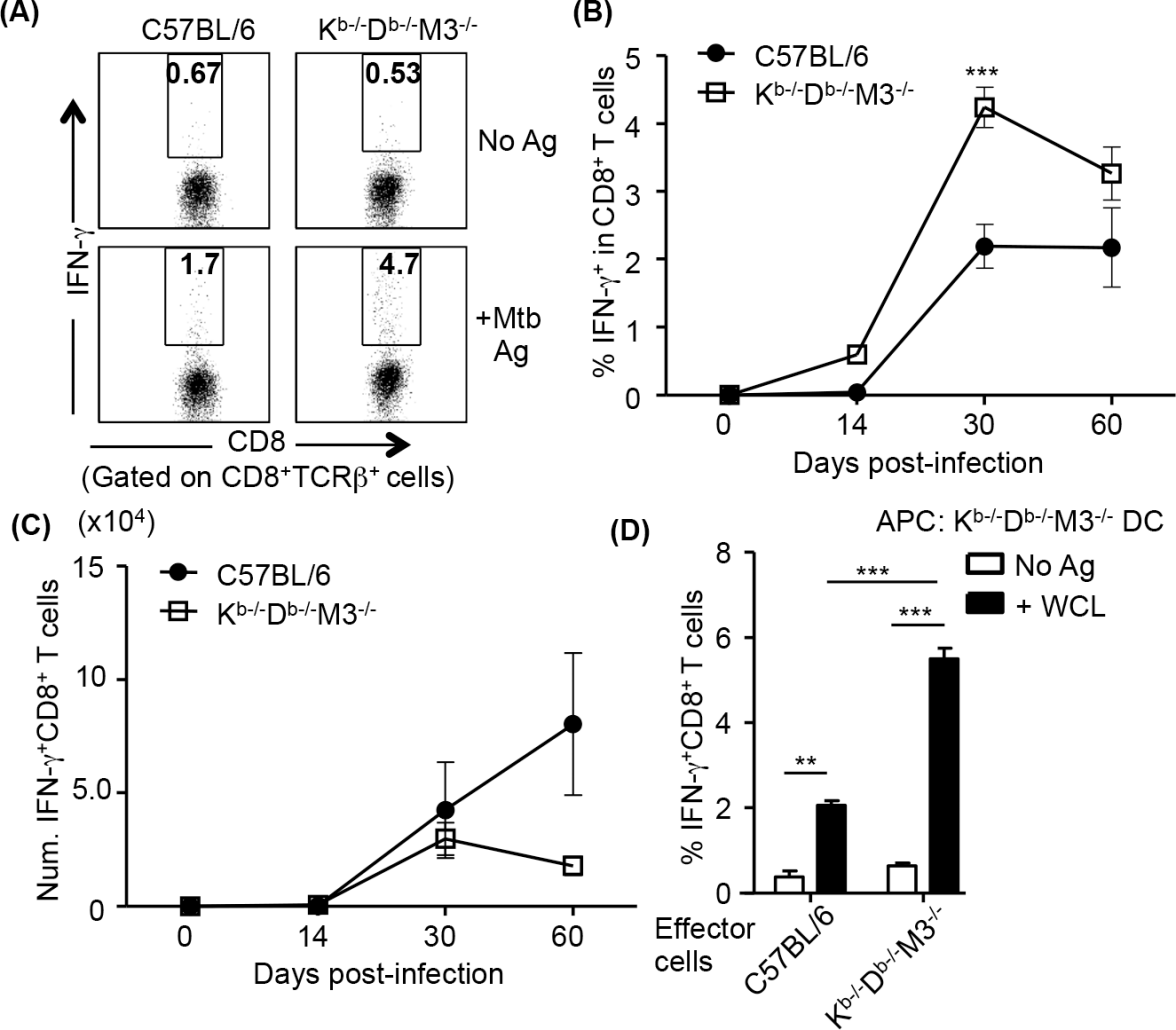
CD8^+^ T cells from Mtb-infected K^b-/-^D^b-/-^M3^-/-^ mice mount a more robust Mtb antigen-specific IFN-γ response as compared to CD8^+^ T cells from B6 mice. CD8^+^ T cells from the lungs of Mtb-infected C57BL/6 and K^b-/-^D^b-/-^M3^-/-^ mice were stimulated *ex vivo* for 18h with Mtb antigen-pulsed C57BL/6 and K^b-/-^D^b-/-^M3^-/-^ BMDCs, respectively, and harvested for intracellular staining of IFN-γ. (A) Representative dot-plots of IFN-γ-producing CD8^+^ T cells in the lungs of C57BL/6 and K^b-/-^D^b-/-^M3^-/-^ mice at day 30 after infection upon stimulation with Mtb whole cell lysates (WCL)-pulsed BMDCs. (B, C) The changes of percentage (B) and total number (C) of WCL-specific CD8^+^ T cells detected in the lungs of K^b-/-^D^b-/-^M3^-/-^ (n = 3–9) and C57BL/6 (n = 3–7) mice during the course of infection. (D) Comparison of the magnitude of Mtb-specific MHC Ib-restricted CD8^+^ T cell responses in C57BL/6 and K^b-/-^D^b-/-^M3^-/-^ mice. CD8^+^ T cells from the lung of C57BL/6 and K^b-/-^D^b-/-^M3^-/-^ mice were stimulated *ex vivo* with WCL-pulsed or un-pulsed K^b-/-^D^b-/-^M3^-/-^ BMDCs for 18h, and harvested for intracellular staining of IFN-γ. Bar graphs depict the mean ± SEM of the percentage IFN-γ-producing CD8^+^ T cells in C57BL/6 (n = 3) and K^b-/-^D^b-/-^M3^-/-^ (n = 5) mice at day 30 post-infection. Data shown are pooled from two independent experiments. **P <0.01, ***P <0.001.

To compare the magnitude of Mtb antigen-specific CD8^+^ T cell responses in B6 and K^b-/-^D^b-/-^M3^-/-^ mice, we isolated lymphocytes from the lungs of B6 and K^b-/-^D^b-/-^M3^-/-^ mice at different time points after infection and determined the number of IFN-γ-producing CD8^+^ T cells upon stimulation with Mtb whole cell lysate (WCL)-pulsed B6 and K^b-/-^D^b-/-^M3^-/-^ BMDCs, respectively. CD8^+^ T cells in the lungs of K^b-/-^D^b-/-^M3^-/-^ and B6 mice at day 30 after infection both produced IFN-γ in response to stimulation with Mtb WCL (Fig 3A). However, the percentage of CD8^+^ T cells producing IFN-γ from Mtb-infected K^b-/-^D^b-/-^M3^-/-^ mice was substantially higher than that seen among CD8^+^ T cells from B6 mice (Fig 3A and 3B). Consequently, there was no significant difference in the absolute number of Mtb-specific IFN-γ producing CD8^+^ T cells between K^b-/-^D^b-/-^M3^-/-^ and B6 mice during the course of Mtb infection (Fig 3C). These results further support the notion that MHC Ib-restricted CD8^+^ T cell responses can compensate for the lack of MHC Ia-restricted CD8^+^ T cell responses during Mtb infection. Importantly, CD8^+^ T cells from Mtb-infected B6 mice were able to produce IFN-γ when stimulated with K^b-/-^D^b-/-^M3^-/-^ BMDCs (Fig 3D), suggesting that Mtb-specific MHC Ib-restricted CD8^+^ T cells are induced in B6 mice after Mtb infection.

### Non-M3, MHC Ib-restricted CD8^+^ T cells recognize Mtb-derived protein antigens in a β_2_m-dependent and TAP-independent manner

**Fig 4:**
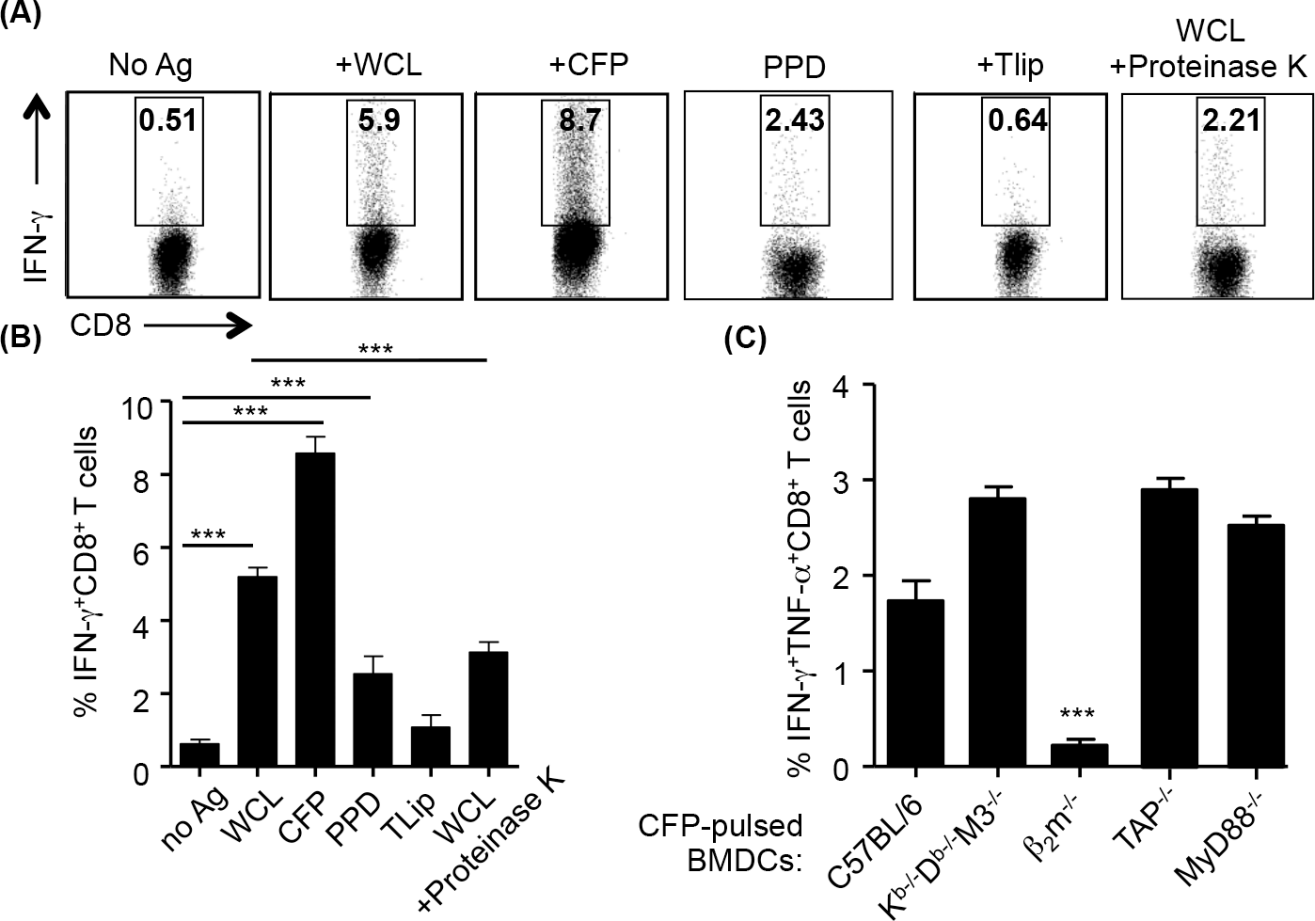
Non-M3, MHC Ib-restricted CD8^+^ T cells recognize Mtb protein antigens in a β_2_m-dependent and TAP-independent manner. CD8^+^ T cells from the lung of K^b-/-^D^b-/-^M3^-/-^ mice at day 30 after infection were stimulated with BMDCs pulsed with or without Mtb antigens, followed by ICS for IFN-γ. (A) Representative dot-plots of IFN-γ-producing CD8^+^ T cells detected from K^b-/-^D^b-/-^M3^-/-^ mice upon stimulation with K^b-/-^D^b-/-^M3^-/-^ BMDCs pulsed with Mtb whole cell lysate (WCL), culture filtrate proteins (CFP), purified protein derivatives (PPD), total lipids (Tlip), and proteinase K-treated WCL, respectively. (B) The percentage of IFN-γ-producing MHC Ib-restricted CD8^+^ T cells detected from K^b-/-^D^b-/-^M3^-/-^ (n = 3) mice in response to different Mtb antigen fractions. (C) Percentage of IFN-γ^+^TNF-α^+^CD8^+^ T cells detected upon stimulation with CFP-pulsed BMDCs from C57BL/6, K^b-/-^D^b-/-^M3^-/-^, β_2_m^-/-^, TAP^-/-^, MyD88^-/-^ mice, respectively. Shown are the percentages of cytokine-positive cells in responses to CFP-pulsed DCs minus the percentage of cytokine-positive cells in responses to the corresponding un-pulsed DCs. Data shown are representative of three independent experiments. ***P <0.001.

Previous studies have shown that different MHC Ib molecules present different types of antigens. For instance, H2-M3, Qa-1/HLA-E and Qa-2 molecules were shown to present peptide antigens [30, 36, 37] while MR1 and CD1d present vitamin B metabolites and lipid antigens, respectively [18, 19, 38]. To determine what kinds of antigens MHC Ib-restricted CD8^+^ T cells from K^b-/-^D^b-/-^M3^-/-^ mice recognize during Mtb infection, CD8^+^ T cells isolated from the lung of Mtb-infected K^b-/-^D^b-/-^M3^-/-^ mice were stimulated with BMDCs pulsed respectively with Mtb WCL, culture filtrate proteins (CFP) and purified protein derivatives (PPD) and total lipids. We expected that the Mtb lipid fraction would contain antigens presented by CD1d while WCL would contain antigens presented by various MHC Ib molecules, including MR1. Eighteen hours after stimulation with the different Mtb antigen preparation, cytokine production was detected by intracellular cytokine staining (ICS). As shown in Fig 4, MHC Ib-restricted CD8^+^ T cells from Mtb-infected K^b-/-^D^b-/-^M3^-/-^ mice produced IFN-γ in response to WCL, PPD and CFP stimulation but not total lipids (Fig 4A and 4B). Meanwhile, when stimulated with proteinase K-treated WCL, the frequency of IFN-γ producing CD8^+^ T cells was reduced by more than 50% (Fig 4A and 4B), suggesting that the majority of Mtb-specific MHC-Ib-restricted CD8^+^ T cells recognize proteinase K-sensitive antigens. As CFP induced a stronger cytokine response than WCL in CD8^+^ T cells from Mtb-infected K^b-/-^D^b-/-^M3^-/-^ mice, we used CFP as antigens in following experiments to study the properties of Mtb-specific MHC-Ib-restricted CD8^+^ T cells.

To determine whether β_2_m or TAP is required for the presentation of Mtb antigens to MHC Ib-restricted CD8^+^ T cells, we stimulated CD8^+^ T cells from Mtb-infected K^b-/-^D^b-/-^M3^-/-^ mice with CFP-pulsed BMDCs derived from B6, K^b-/-^D^b-/-^M3^-/-^, TAP^-/-^ and β_2_m^-/-^ mice and measured cytokine production by ICS. We observed a significant reduction in the percentage of IFN-γ^+^TNF-α^+^CD8^+^ T cells upon stimulation with β_2_m^-/-^ BMDCs compared to stimulation with B6 or K^b-/-^D^b-/-^M3^-/-^ BMDCs (Fig 4C). In contrast, no reduction in the percentage of cytokine-producing cells was observed when stimulated with BMDCs from TAP^-/-^ mice (Fig 4C). These data indicated that MHC Ib-restricted CD8^+^ T cells recognize Mtb protein antigens in a β_2_m-dependent and TAP-independent manner. As some MHC Ib-restricted T cells can be activated by pro-inflammatory cytokines in an antigen-independent manner [39, 40], we also examined whether MyD88-mediated signaling affected cytokine-production by CD8^+^ T cells from Mtb-infected K^b-/-^D^b-/-^M3^-/-^ mice. A similar percentage of cytokine-producing CD8^+^ T cells was observed upon stimulation with MyD88-deficient BMDCs compared to stimulation with K^b-/-^D^b-/-^M3^-/-^ BMDCs (Fig 4C), suggesting that MyD88 pathway likely does not play a major role in the activation of MHC Ib-restricted CD8^+^ T cells.

### Non-M3, MHC Ib-restricted CD8^+^ T cells exhibit polyfunctional characteristics

**Fig 5:**
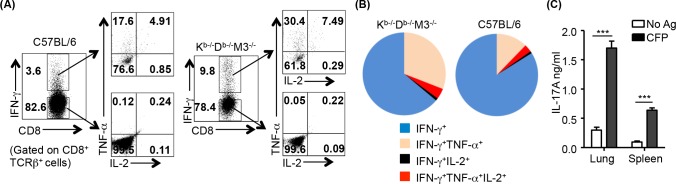
Non-M3, MHC Ib-restricted CD8^+^ T cells exhibit polyfunctional characteristics. CD8^+^ T cells from the lung or spleen of C57BL/6 and K^b-/-^D^b-/-^M^3-/-^ mice were stimulated *ex vivo* for 18h with Mtb antigen-pulsed C57BL/6 and K^b-/-^D^b-/-^M3^-/-^ BMDCs, respectively, then harvested for intracellular staining of IFN-γ, TNF-α and IL-2. (A) Representative dot-plots of multiple cytokine-producing CD8^+^ T cells in the lung of C57BL/6 and K^b-/-^D^b-/-^M3^-/-^ mice at day 30 after infection upon stimulation with Mtb culture filtrate proteins (CFP). (B) Pie graphs show relative proportion of TNF-α^+^, IL-2^+^ and TNF-α^+^IL-2^+^ CD8^+^ T cells among IFN-γ^+^CD8^+^ T cells from the lung of Mtb-infected K^b-/-^D^b-/-^M3^-/-^ and C57BL/6 mice. (C) IL-17A production from enriched CD8^+^ T cells in Mtb-infected K^b-/-^D^b-/-^M3^-/-^ mice detected by ELISA after stimulation with CFP. ***P <0.001.

Polyfunctional T cells simultaneously producing multiple cytokines (IFN-γ, TNF-α and IL-2) have been shown to either correlate with protective immunity against Mtb [41, 42] or TB disease activity in humans [43, 44]. To examine whether MHC Ib-restricted CD8^+^ T cells displayed multi-functionality during Mtb infection, CD8^+^ T cells isolated from the lungs of Mtb-infected K^b-/-^D^b-/-^M3^-/-^ mice were stimulated with CFP-pulsed BMDCs, and responses were analyzed using ICS for IFN-γ, TNF-α and IL-2. We found K^b-/-^D^b-/-^M3^-/-^ mice had a higher percentage of double-cytokine-producing (IFN-γ^+^TNF-α^+^) and triple-cytokine-producing (IFN-γ^+^TNF-α^+^IL-2^+^) CD8^+^ T cells compared to B6 mice (Fig 5A and 5B). In addition, enriched CD8^+^ T cells from lungs and spleens of Mtb-infected K^b-/-^D^b-/-^M3^-/-^ mice also produced IL-17A in the presence of CFP (Fig 5C). Taken together, these results indicate that after Mtb infection, the MHC Ib-restricted CD8^+^ T cell responses in K^b-/-^D^b-/-^M3^-/-^ mice are antigen-specific and polyfunctional.

### Non-M3, MHC Ib-restricted CD8^+^ T cells recognize various Mtb protein antigens

**Fig 6:**
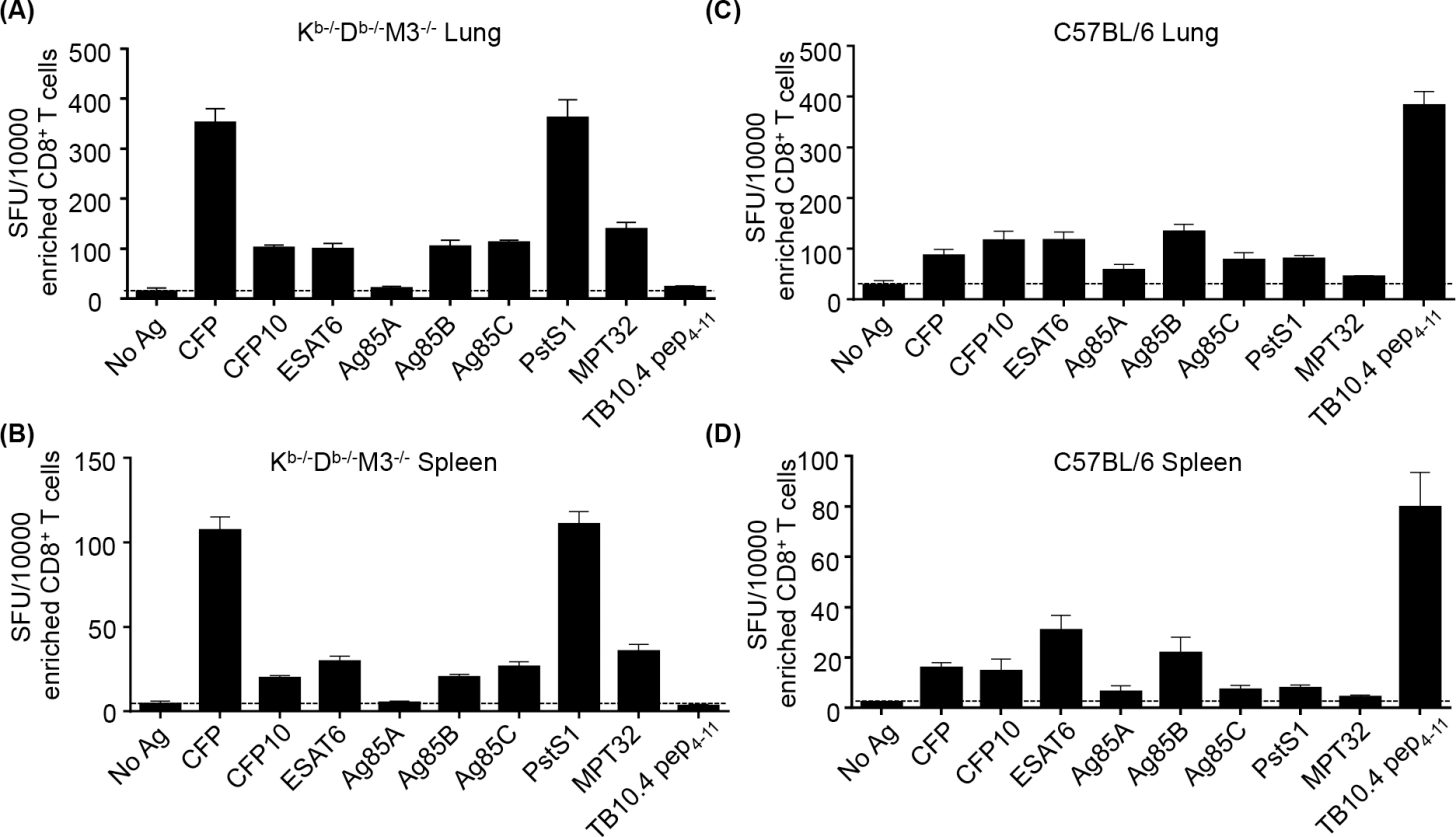
Non-M3, MHC Ib-restricted CD8^+^ T cells recognize various Mtb antigens. At day 30 post-infection, CD8^+^ T cells from Mtb-infected K^b-/-^D^b-/-^M3^-/-^ and B6 mice were used in ELISPOT assays using various Mtb antigen-pulsed K^b-/-^D^b-/-^M3^-/-^ or B6 BMDCs as stimulators. (A, B) The frequency of IFN-γ-producing CD8^+^ T cells detected in the lung (A) and spleen (B) of K^b-/-^D^b-/-^M3^-/-^ mice (n = 3) in response to *in vitro* stimulation with various Mtb antigens. (C, D) The frequency of IFN-γ-producing CD8^+^ T cells detected in the lung (C) and spleen (D) of B6 mice (n = 3) upon stimulation with various Mtb antigens. Dot lines depict the background cytokine production in the absence of Mtb Ag. Data shown are representative of three independent experiments.

Several immunodominant antigens recognized by Mtb-specific MHC Ia-restricted CD8^+^ T cells have been identified [10]. However, little is known about the Mtb antigens recognized by MHC Ib-restricted CD8^+^ T cells. To address this issue, we examined the reactivity of CD8^+^ T cells in Mtb-infected K^b-/-^D^b-/-^M3^-/-^ mice to several representative immunogenic Mtb antigens, including CFP, CFP10, ESAT6, Ag85A, Ag85B, Ag85C, PstS1, MPT32 and TB10.4_4−11_ peptide, in an IFN-γ Elispot assay. As shown in Fig 6, enriched CD8^+^ T cells from lungs and spleens of K^b-/-^D^b-/-^M3^-/-^ mice at day 30 post-infection responded to most Mtb antigens tested except Ag85A and the TB10.4_4−11_ peptide, which is known to be restricted by MHC Ia molecule H2-K^b^ [45] (Fig 6A and 6B). The Mtb antigen-specific CD8^+^ T cell responses were higher in the lungs compared to the spleens, but the pattern of reactivity was similar between the organs. Among these Mtb antigen-specific MHC Ib-restricted CD8^+^ T cells, the frequencies of CFP- and PstS1-specific CD8^+^ T cells were highest (Fig 6A and 6B). In contrast, CD8^+^ T cells from B6 mice recognized most of these Mtb antigens at a lower frequency but exhibited strong reactivity to TB10.4_4−11_ peptide (Fig 6C and 6D). These results indicate that MHC Ia and MHC Ib-restricted CD8^+^ T cells recognize distinct Mtb antigens during infection. It also suggests that PstS1 might be an immunodominant antigen recognized by MHC Ib-restricted CD8^+^ T cells.

### The expanded CD8^+^ T cells after Mtb infection include Qa-2-restricted T cells

**Fig 7:**
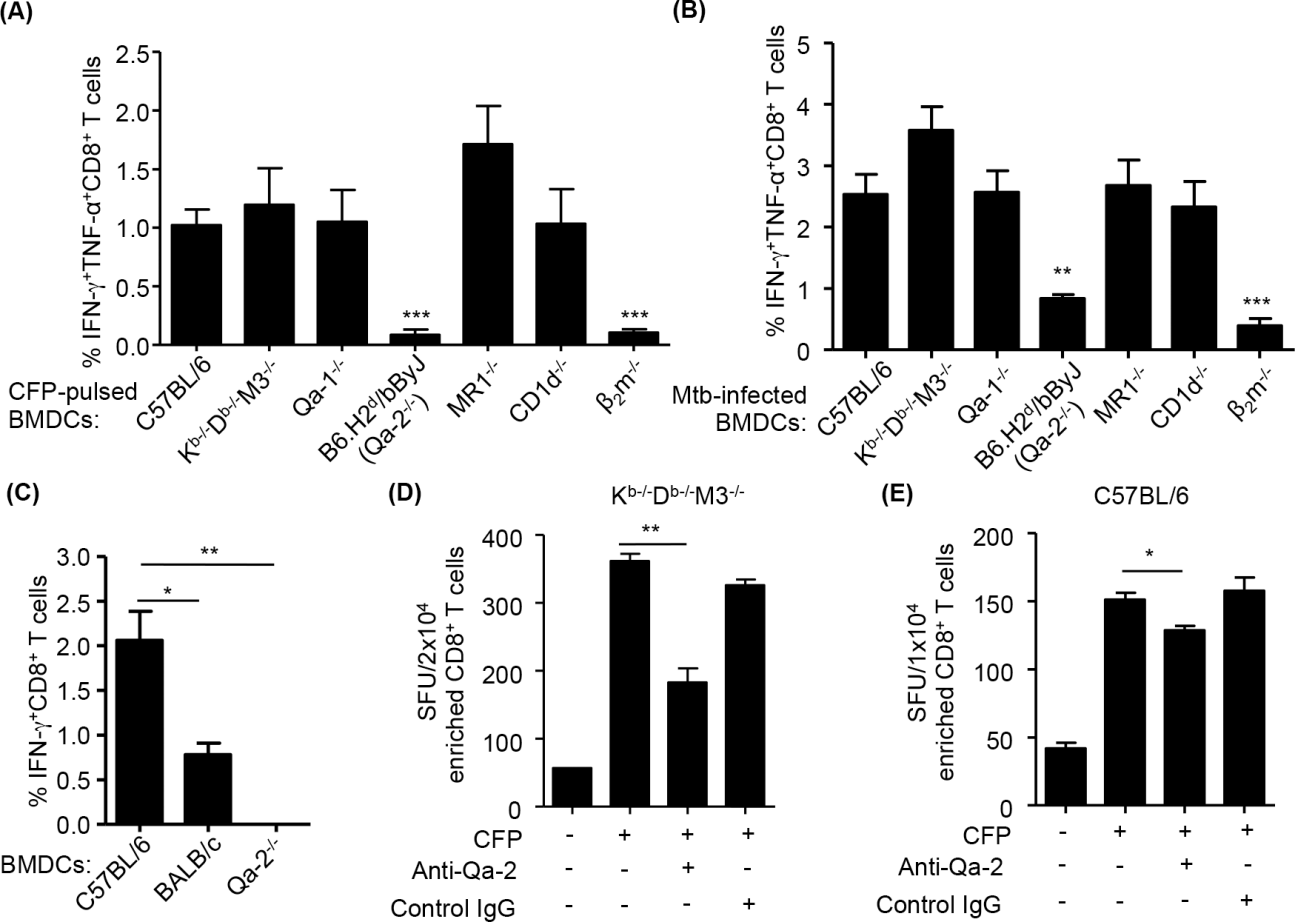
Qa-2-restricted T cells comprise a significant proportion of the expanded CD8^+^ T cells during Mtb infection. CD8^+^ T cells from the lungs of Mtb-infected K^b-/-^D^b-/-^M3^-/-^ mice at day 30 post-infection were stimulated with un-pulsed or CFP-pulsed BMDCs or Mtb-infected BMDCs from indicated mice and intracellularly stained for the cytokine IFN-γ and TNF-α. (A, B) The percentage of cytokine-producing CD8^+^ T cells in response to stimulation with CFP-pulsed (A) and Mtb-infected (B) BMDCs derived from B6 and various MHC Ib molecule-deficient mice. (C) The percentage of IFN-γ^+^CD8^+^ T cells upon stimulation with CFP-pulsed BMDCs that expressed different level of Qa-2. The percentage shown is the percentage of cytokine-producing cells with Ag stimulation minus the baseline without Ag stimulation. (D, E) CD8^+^ T cells from spleens or lungs of K^b-/-^D^b-/-^M3^-/-^ (D) and C57BL/6 (E) mice were stimulated with un-pulsed or CFP-pulsed K^b-/-^D^b-/-^M3^-/-^ BMDCs in the presence of anti-Qa-2 (20-8-4) or control IgG (anti-K^b^, Y3 or MOPC1) and the IFN-γ-secreting cells were quantified in an ELISPOT assay. Data shown are representative of three independent experiments, and are the mean ± SEM (n = 3 per experiment). **P <0.01, ***P <0.001.

To investigate which MHC class Ib molecules might present Mtb antigens to CD8^+^ T cells during infection, we enriched CD8^+^ T cells from K^b-/-^D^b-/-^M3^-/-^ mice and stimulated them with CFP-pulsed as well as Mtb-infected BMDCs derived from various mouse strains (i.e. B6, K^b-/-^D^b-/-^M3^-/-^, Qa-1^-/-^, Qa-2^null^, MR1^-/-^, CD1d^-/-^ and β_2_m^-/-^ mice), and examined their cytokine production by ICS. Compared to stimulation with B6 BMDCs, there was no difference in the percentage of cytokine-producing CD8^+^ T cells when stimulated with BMDCs from Qa-1^-/-^, MR1^-/-^, CD1d^-/-^ and K^b-/-^D^b-/-^M3^-/-^ mice in either the CFP-pulsed or Mtb-infected BMDCs (Fig 7A and 7B). This suggested that most of the cytokine-producing CD8^+^ T cells in K^b-/-^D^b-/-^M3^-/-^ were not restricted by these MHC I molecules. However, when stimulated with BMDCs from Qa-2^null^ mice, the percentage of cytokine-producing CD8^+^ T cells decreased to the same level as seen with β_2_m^-/-^ BMDCs stimulation (Fig 7A and 7B), suggesting that a substantial fraction of Mtb-specific CD8^+^ T cells in K^b-/-^D^b-/-^M3^-/-^ mice are restricted to Qa-2.

To substantiate this finding, we examined the cytokine production of these CD8^+^ T cells in response to stimulation with Mtb antigen-pulsed BMDCs derived from mice that express different levels of Qa-2, i.e. C57BL/6 (Qa-2^hi^), BALB/cJ (Qa-2^lo^), and B6.C-H2^d^/bByJ (Qa-2^null^) [46, 47] (S2 Fig). We found the percentage of Mtb-specific IFN-γ-producing CD8^+^ T cells was higher when CD8^+^ T cells were co-cultured with BMDCs from C57BL/6 than from BALB/cJ mice and almost undetectable when stimulated with Qa-2^null^ BMDCs, indicating that IFN-γ production was correlated to the expression level of Qa-2 on the BMDCs (Fig 7C). Since Qa-2^null^ BMDCs and B6 BMDCs induced a similar IFN-γ response from LemA-specific M3-restricted CD8^+^ T cells (S2 Fig), which suggested that Qa-2^null^ BMDCs are capable of presenting antigens to other MHC Ib-restricted T cells. Furthermore, pretreatment of K^b-/-^D^b-/-^M3^-/-^ BMDCs with an anti-Qa-2 antibody (clone 20-8-4)[48], but not control IgG, resulted in a significant reduction of Mtb-specific IFN-γ secretion by CD8^+^ T cells isolated from Mtb-infected K^b-/-^D^b-/-^M3^-/-^ mice (Fig 7D). Collectively, these results indicate that a large proportion of unconventional Mtb-specific CD8^+^ T cells found in K^b-/-^D^b-/-^M3^-/-^ mice are Qa-2-restricted. To determine if Qa-2-restricted T cell responses can be detected in Mtb-infected B6 mice, we performed Qa-2 blocking experiment in an ELISPOT assay. We found that the percentage of IFN-γ producing CD8^+^ T cells in Mtb-infected B6 mice was significantly reduced when stimulated with CFP-pulsed K^b-/-^D^b-/-^M3^-/-^ BMDCs in the presence of anti-Qa-2 blocking antibody (Fig 7E). This suggested the existence of Mtb-specific Qa-2-restricted T cells in B6 mice following Mtb infection.

### MR1-restricted T cells do not constitute a significant proportion of MHC Ib-restricted T cells in K^b-/-^D^b-/-^M3^-/-^ mice after Mtb infection

**Fig 8:**
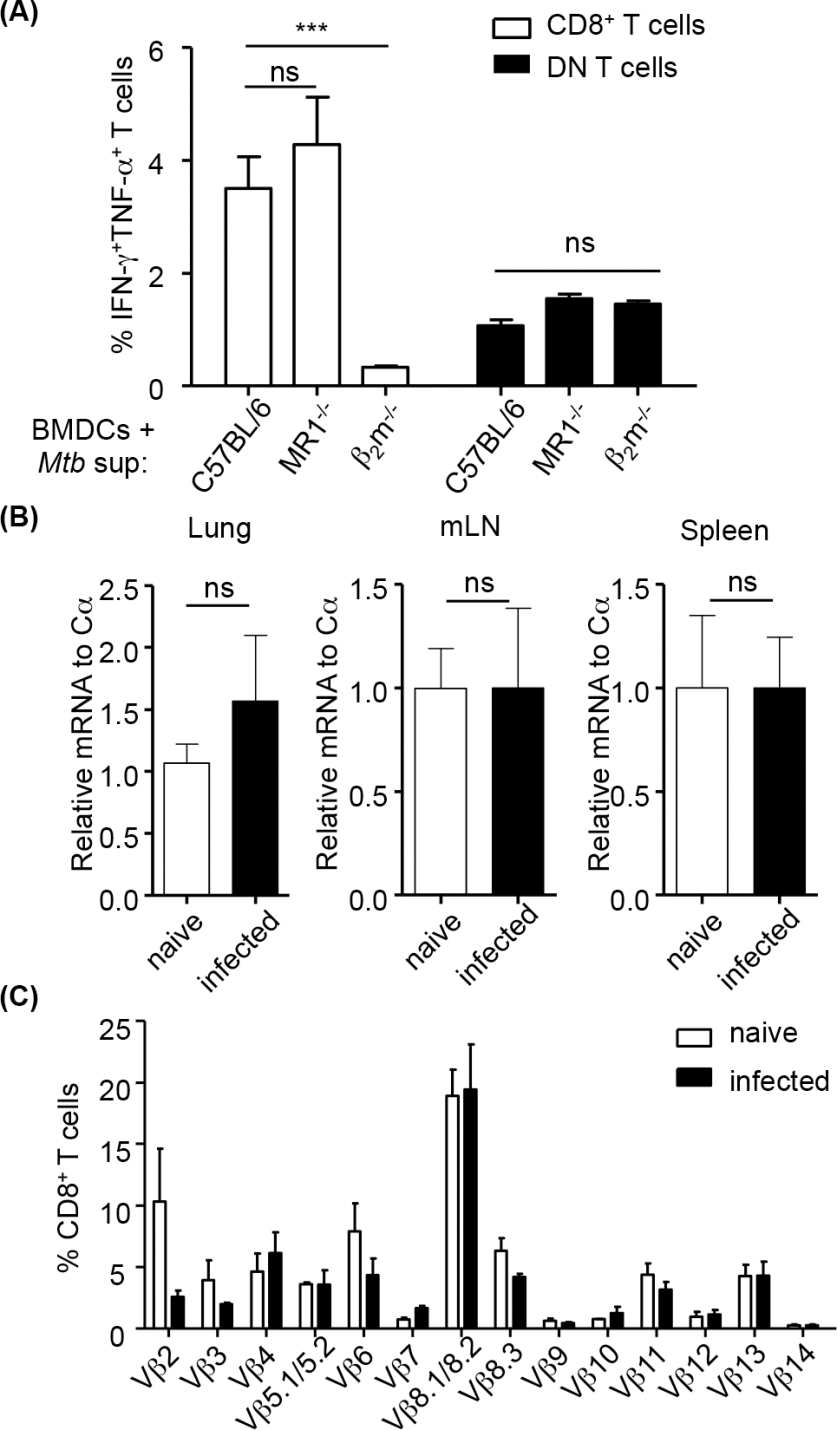
MR1-restricted T cells do not represent a significant population of Mtb-specific T cells in K^b-/-^D^b-/-^M3^-/-^ mice after Mtb infection. (A) Lymphocytes isolated from the lungs of Mtb-infected K^b-/-^D^b-/-^M3^-/-^ mice were stimulated with Mtb culture supernatant-pulsed BMDCs from C57BL/6, MR1^-/-^ and β_2_m^-/-^ mice. Percentages of cytokine-producing CD8^+^ and CD4^-^CD8^-^ (DN) T cells were determined by intracellular cytokine staining. Data are representative of two independent experiments, and are the mean ± SEM. (n = 3). ***P<0.001. (B) CD8^+^ T cells isolated from the lung, mediastinal lymph node and spleen of naïve and Mtb-infected K^b-/-^D^b-/-^M3^-/-^ mice (at day 30 post-infection) were used to examine the expression of Vα19-Jα33 transcripts by quantitative RT-PCR (n = 6 per group). qPCR results are presented as relative units normalized to TCRα constant region mRNA. ns, no statistical significance. (C) TCR Vβ usage of CD8^+^ T cells in naïve and Mtb-infected K^b-/-^D^b-/-^M3^-/-^ mice (n = 5) at day 30 post-infection.

A recent study showed that MR1^-/-^ mice had higher bacterial load in the lung compared to wild-type mice after BCG infection [26], suggesting a role for MAIT cells during mycobacteria infection. In addition, MAIT cells were found to be highly enriched in the bronchoalveolar lavage fluid of patients with active TB [49]. Although we did not detect significant number of Mtb-specific MR1-restricted T cells when we stimulated CD8^+^ T cells from Mtb-infected K^b-/-^D^b-/-^M3^-/-^ mice with Mtb-infected BMDCs (Fig 7B), it remains possible that *in vitro* Mtb-infected BMDCs do not contain sufficient amounts of MR1 ligands. To address this issue, culture supernatants from logarithmic phase of Mtb culture (Mtb sup) were used as antigen(s) since they contain vitamin B metabolites known to activate MAIT cells [38, 50]. As both CD8^+^ and CD4^-^CD8^-^ (DN) MAIT cells are present in the lung of naïve mice [51], we stimulated CD8^+^ and DN T cells from Mtb-infected K^b-/-^D^b-/-^M3^-/-^ mice with Mtb sup-pulsed MR1^-/-^, β_2_m^-/-^ and B6 BMDCs, and measured cytokine production by ICS. We found that IFN-γ and TNF-α production by CD8^+^ T cells was β_2_m-dependent but not MR1-dependent, while the cytokine production by DN T cells was neither MR1-dependent nor β_2_m-dependent (Fig 8A). These results suggest that MAIT cells do not constitute a significant proportion of CD8^+^ and DN T cells expanded in K^b-/-^D^b-/-^M3^-/-^ mice during Mtb infection. Consistent with this finding, no significant differences in the expression of Vα19-Jα33 mRNA, the canonical TCR α chain of MAIT cells [52], were detected in the lungs, spleens and mediastinal lymph nodes at day 30 after Mtb infection, compared to naïve mice (Fig 8B). Furthermore, unlike MAIT cells, which preferentially use Vβ6 or Vβ8, MHC Ib-restricted CD8^+^ T cells found in Mtb-infected K^b-/-^D^b-/-^M3^-/-^ mice use diverse TCR Vβ chains (Fig 8C). Taken together, our data suggest that MAIT cells do not represent a significant population of MHC-Ib restricted T cells in the mouse model of TB infection.

## Discussion

In this study, we defined the relative contribution of MHC Ia-, M3- and other MHC Ib-restricted CD8^+^ T cells in Mtb infection by comparing CD8^+^ T cell responses in B6, K^b-/-^D^b-/-^, K^b-/-^D^b-/-^M3^-/-^ and β_2_m^-/-^ mice upon aerosol infection with virulent Mtb. Unlike their role in *Listeria* infection, M3-restricted CD8^+^ T cells do not play a dominant role in the MHC Ib-restricted CD8^+^ T cell responses to Mtb infection. This finding highlights the differential roles of various MHC Ib-restricted responses in immunity against distinct microbial pathogens. While CD1-restricted and MR1-restricted T cell responses have been characterized in the context of Mtb infection, our data showed that neither CD1d nor MR1 serve as major restriction elements for the Mtb-specific MHC Ib-restricted CD8^+^ T cells found in K^b-/-^D^b-/-^M3^-/-^ mice. In fact, we found a substantial fraction of these Mtb-specific unconventional CD8^+^ T cells were restricted by Qa-2, which is known to present a more diverse array of peptides than other MHC Ib molecules [53]. Qa-2-restricted T cell responses have been implicated in anti-tumor immunity [48] and antiviral immunity [37] [54]. However, this is the first study to describe a role for Qa-2 in host defense against bacterial infection. As HLA-G is a possible functional homolog of Qa-2 [55], it will be of great interest to explore whether HLA-G-restricted Mtb-specific T cell responses can be detected in patients with active TB or BCG-vaccinated individuals.

Previous studies have shown that K^b-/-^D^b-/-^ mice were more resistant to Mtb infection than β_2_m^-/-^ mice [31, 32]. However, these studies did not address whether CD8^+^ T cells in Mtb-infected K^b-/-^D^b-/-^mice contribute to β_2_m-dependent resistance to Mtb-infection. The observation from our study that *in vivo* depletion of CD8^+^ T cells in K^b-/-^D^b-/-^M3^-/-^ mice resulted in increased susceptibility to Mtb infection, clearly demonstrates a role for non-M3, MHC Ib-restricted CD8^+^ T cells in the control of Mtb infection. It has been shown that some TCRβ^+^ CD4^-^CD8^-^ (DN) T cells or TCRγδ^+^ T cells recognize MHC Ib molecules. However, we did not detect significant expansion of γδ T cells in K^b-/-^D^b-/-^M3^-/-^ mice after Mtb infection (S3 Fig). In addition, we found that the expansion of DN T cells in Mtb-infected mice was largely β_2_m-independent (S4 Fig), suggesting that MHC Ib molecules do not play a critical role in the expansion of DN T cells during Mtb infection. Thus, the major contribution of MHC-Ib molecules in Mtb infection is likely mediated through MHC Ib-restricted CD8^+^ T cells. In contrast to a previous report [31], we did not observe significant differences in bacterial burdens between B6 and K^b-/-^D^b-/-^ mice following aerogenic Mtb infection at the time points examined. This discrepancy could in part due to the use of different backcross generations of K^b-/-^D^b-/-^ mice (i.e. 6 times backcrossed versus 10 times backcrossed) and/or animal housing environments.

Unlike MHC class Ia-restricted CD8^+^ T cells, MHC class Ib-restricted CD8^+^ T cells in naïve mice exhibit activated/memory T cell phenotype that is CD44^hi^, CD11a^hi^, CD122^hi^ and Ly6C^hi^ [31, 56]. Our labs and others have shown that MHC class Ib-restricted CD8^+^ T cells rapidly respond to *Listeria* infection [57, 58]. Similarly, during Mtb infection, though the frequency of CD8^+^ T cells in naïve K^b-/-^D^b-/-^M3^-/-^ mice was much lower than that in naïve B6 mice, we eventually detected similar number of effector CD8^+^ T cells (Fig 2B). In addition, we detected comparable number of IFN-γ-producing CD8^+^ T cells (Fig 3C) between Mtb-infected K^b-/-^D^b-/-^M3^-/-^ and B6 mice upon *ex vivo* stimulation with Mtb antigens. These results suggest that MHC Ib-restricted CD8^+^ T cells mount a more robust response than conventional CD8^+^ T cells following Mtb infection. It is noteworthy that Mtb-specific MHC Ib-restricted CD8^+^ T cell responses can be readily detected in the lung of B6 mice following Mtb infection, albeit the magnitude is lower compared to those observed in Mtb-infected K^b-/-^D^b-/-^M3^-/-^ mice. Therefore, it is possible that the presence of MHC Ia-restricted CD8^+^ T cells could affect the precursor frequency and peripheral expansion of MHC Ib-restricted CD8^+^ T cells.

Memory-like CD44^hi^ CD8^+^ T cells [39] and innate-like T cells [40] have been shown to be activated either through the engagement of TCR with microbial antigens or cytokine-driven signals during infection. Some of the Mtb antigens used in our study could enhance the production of inflammatory cytokines by BMDCs in part through the MyD88-dependent pathway (S5 Fig). However, we found CD8^+^ T cells isolated from Mtb-infected K^b-/-^D^b-/-^M3^-/-^ mice recognize proteinase K-sensitive antigens and their activation is independent of MyD88-mediated signaling, suggesting these MHC Ib-restricted CD8^+^ T cells are activated through TCR recognition of Mtb-derived antigens. This notion is further supported by our results, which showed MHC Ib restricted CD8^+^ T cells produced IFN-γ in response to stimulation with several recombinant Mtb protein antigens. Nevertheless, it remains possible that some MHC Ib-restricted CD8^+^ T cells can be activated *in vivo* via a bystander mechanism during Mtb infection.

Several studies showed that antigens present in Mtb culture filtrate proteins are highly effective in inducing protective immunity against Mtb infection [10]. We found that non-M3 MHC Ib restricted CD8^+^ T cells responded to several of these antigens. Among the Mtb antigens tested, the phosphate-binding transporter lipoprotein PstS1 seems to induce the strongest response. In contrast, CD8^+^ T cells from Mtb-infected B6 mice exhibited strong reactivity to TB10.4_4−11_ peptide, but had a weaker response to other Mtb CFP antigens tested. These data suggest that immunodominant antigens targeted by MHC Ia and MHC Ib-restricted CD8^+^ T cells are substantially different. Mtb antigen-specific CD8^+^ T cell responses detected in K^b-/-^D^b-/-^M3^-/-^ mice were TAP-independent. As a large proportion of these Mtb-specific MHC Ib-restricted T cells are Qa-2-restricted CD8^+^ T cells, it is possible that Qa-2 might present Mtb antigens in a TAP-independent manner, similar to HLA-E in humans [29, 30, 59]. However, it is not clear whether the TAP-independent pathway is preferentially used by Mtb-infected cells presenting antigens to MHC Ib-restricted CD8^+^ T cells *in vivo*.

In an effort to further define the role of Qa-2 during Mtb infection, we infected two sub-strains of BALB/c mice, BALB/cJ (Qa-2-sufficient) and BALB/cByJ (Qa-2-deficient), with Mtb and compared the bacterial burden and Mtb-specific T cell responses between these two sub-strains. We found that BALB/cByJ mice lacked Mtb-specific Qa-2-restricted CD8^+^ T cells and had a higher bacterial burden in the spleen compared to BALB/cJ mice (S6 Fig). These results suggest that Mtb-specific Qa-2-restricted CD8^+^ T cells contribute to protection against Mtb infection. However, it is known that BALB/cByJ and BALB/cJ mice have additional genetic differences besides Qa-2 expression. Thus, further experiments involving Qa-2 knockout mice in the B6 background (this mouse strain is yet to be generated) are needed to definitively confirm the role of Qa-2-restricted CD8^+^ T cells during Mtb infection.

In summary, TB remains a challenge for human health and protective cellular immunity is extremely desirable for the design of better Mtb vaccines. Our finding that nonclassical CD8^+^ T cells, largely Qa-2 restricted CD8^+^ T cells, can provide protection against Mtb revealed the presence of a potentially novel cellular population in combating tuberculosis. In humans, a large proportion of Mtb-specific CD8^+^ T cells appear to be restricted by MHC Ib molecules [60]. Targeting these MHC Ib-restricted CD8^+^ T cells would facilitate the design of better vaccines against Mtb that can induce broader immune protection than targeting MHC Ia-restricted CD8^+^ T cells in genetically diverse human populations due to the limited polymorphism of MHC class Ib molecules.

## Materials and Methods

### Ethics statement

This study was carried out in strict accordance with the recommendations in the Guide for the Care and Use of Laboratory Animals of the National Institutes of Health. The protocol was approved by the Animal Care and Use Committee of the Northwestern University (Protocol number: IS00000985).

### Mice

K^b-/-^D^b-/-^, K^b-/-^D^b-/-^M3^-/-^ [56], and CD1d^-/-^ [61] on the B6 background mice were generated or maintained in house. C57BL/6, BALB/cJ, BALB/cByJ, TAP^-/-^, β_2_m^-/-^ and B6.C-H2^d^/bByJ mice (Qa-2 ^null^) [62] were purchased from The Jackson Laboratory (Bar Harbor, ME). MyD88^-/-^ mice were obtained from Mutant Mouse Resource and Research Centers. MR1^-/-^ [63], Qa-1^b-/-^ [64] mice were provided by Dr. Ted Hansen (Washington University School of Medicine, St Louis, MO) and by Dr. Harvey Cantor (Dana-Farber Cancer Institute, Boston, MA), respectively.

### Mtb fractions and antigens

Mtb fractions and recombinant Mtb proteins (CFP10, PstS1, Ag85A, Ag85B, Ag85C, MPT32 and ESAT6) were obtained through BEI Resources (Manassas, Virginia). Mtb fractions and antigens were dissolved in either DMSO or PBS and stored as aliquots at -20°C.

### Mtb aerosol infection and CFU counting

For Mtb aerosol infection, frozen aliquots of Mtb H37Rv were thawed and diluted in PBS with 0.05% Tween 80. Mice were infected with 100–200 CFU using a nose-only aerosol exposure chamber (In-Tox Products, NM), equipped with a Lovelace nebulizer, as previously described [24]. A day 1 count was performed to determine the infecting dose. At indicated time-points after infection, bacterial loads in lungs and spleens were determined by plating serial dilutions of homogenate on Middlebrook 7H11 agar plates (BBL, BD), and colonies were counted after 2–3 weeks of incubation at 37°C.

### Primary cell preparation and dendritic cell generation

Single-cell suspensions were prepared from the lung, spleen and mediastinal lymph node by mechanical disruption in HBSS/2% FBS. Lung was digested with collagenase IV (1mg/ml) and DNase I (30μg/ml) for 30 min at 37°C before disruption. To enrich CD8^+^ T cells, splenocytes and lung leukocytes were labeled with biotinylated mAb specific to CD19 (6D5), CD4 (GK1.5), CD11b (M1/70), CD49b (DX5), TCRγδ (GL-3) and I-A/I-E^b^ (M5/114.15.2) (Biolegend, San Diego, CA) followed by streptavidin-conjugated magnetic beads (Dynabeads, Invitrogen). The purity and composition of enriched CD8^+^ T cells were confirmed by flow cytometry. Bone marrow-derived dendritic cells (BMDCs) were derived from mouse bone marrow progenitors using GM-CSF and IL-4 (PeproTech, Rocky Hill, NJ) as previously described [56].

### *In vitro* infection of BMDCs with Mtb H37Rv

BMDCs were plated in 96-well flat-bottom plate at a centration of 1x10^5^ cells/well and infected at a multiplicity of 1 or 3 with Mtb for 2h at 37°C. Cultures were washed three times and treated with 20 μg/ml gentamycin for 2 h to remove extracellular bacteria. Enriched CD8^+^ T cells from Mtb-infected mice were added 24h later to the indicated wells for co-culture. To determine bacterial uptake, some BMDCs were lysed with sterile distilled water containing 0.01% SDS and plated on 7H11 plate.

### Antibodies and flow cytometry

Monoclonal antibodies against mouse CD8α (53–6.7), CD8β (YTS156.7.7), CD44 (1M7), CD62L (MEL14), CD127 (A7R34), PD-1 (29F.1A12), KLRG1 (2F1/KLRG1), B220 (RA36B2), CD4 (GK 1.5), TCRβ (H57-597), Gr-1 (1A8), NK1.1 (PK136), anti-Qa-2 (1-1-2), Vβ2 (B20.6), Vβ3 (KJ25), Vβ4 (KT4), Vβ5.1/5.2 (MR9-4), Vβ6 (RR4-7), Vβ7 (TR310), Vβ8.1/8.2 (MR5-2), Vβ8.3 (1B3.3), Vβ9 (MR10-2), Vβ10 (B21.5), Vβ11 (RR3-15), Vβ12 (MR11.1), and Vβ13 (MR12-3) with different fluorochrome conjugate, were purchased either from BioLegend or eBioscience or BD Bioscience (San Diego, CA). Anti-Qa-2 mAb (20-8-4) was purified from hybridoma culture supernatant using the protein A column. For cell surface staining, cells were incubated with 2.4G2 Fcγ RII/RIII blocking mAb for 15 min, then stained with the appropriate combinations of mAbs in HBSS/2% FBS for 30 min at 4°C. Flow cytometry was performed with a FACS CantoII (BD Biosciences, San Jose, CA) and analyzed using FlowJo software (Tree Star, Ashland, OR).

### Intracellular cytokine staining

T cells or CD8^+^ T cells from the spleen and lung of infected mice were stimulated with unpulsed or Mtb antigen-pulsed BMDCs or Mtb-infected BMDCs. After two hours of incubation, Brefeldin A (10μg/ml, Sigma, St. Louis, MO) was added and cells were cultured for additional 16 hours. After incubation period, cells were harvested, stained for cell surface markers, fixed with 4% paraformaldehyde, permeabilized with 0.2% saponin and stained with APC-conjugated anti-IFN-γ (eBioscience), FITC-conjugated anti-TNFα (Biolegend) and PE-conjugated anti-IL-2 mAbs (eBioscience). Flow cytometry was performed as described earlier.

### ELISPOT assay

IFN-γ Elispot assay was performed as previously described [24], with some modifications. Briefly, multiscreen-IP plates (Millipore, Bedford, MA) were coated with anti-IFN-γ mAb (An-18, eBioscience) at 5μg/ml in PBS. BMDCs were pre-pulsed with Mtb fractions or recombinant protein antigens overnight. In a blocking assay, Mtb antigen-pulsed BMDCs were pre-incubated with mouse IgG or anti-Qa-2 mAb (20-8-4) [48] for 30 min before the assay. Enriched CD8^+^ T cells from infected mice (5×10^3^−2×10^4^) were mixed with BMDCs stimulator cells (5×10^4^/well) in RPMI 10 medium and plated in triplicate wells. After 18h incubation at 37°C, plates were washed using PBS-Tween (PBS and 0.05% Tween 20) and incubated for 2h at room temperature with biotinylated anti-IFN-γ mAb (R4.6A2, eBioscience). Plates were then washed and incubated with streptavidin-conjugated alkaline phosphatase (Jackson ImmunoResearch Laboratories, West Grove, PA). After 1 h incubation at room temperature, plates were developed with a BCIP/NBT substrate kit (Bio-Rad, Hercules, CA) according to the manufacturer’s instructions. Spots were counted using an ImmunoSpot reader (Cellular Technology, Shaker Heights, OH).

### In vivo depletion of CD8^+^ T cells

Mice were given 100 μg of anti-CD8β mAb (53–5.8) or normal rat IgG (controls) by the i.p. route on days -3, 0, 7, 14, and 21 post infection with Mtb, as described by Wang et al.[65]. Mice were killed on day 28 post infection and the depletion efficiency was determined by flow cytometry.

### Quantitative RT-PCR

Total RNA was extracted using Trizol reagent (Invitrogen), and first-strand cDNA was synthesized using the Superscript III reverse transcriptase (Invitrogen) according to the manufacturer’s instructions. Real-time PCR was performed by using IQ5 instrument (Bio-Rad systems). The expression of *V*α*19-J*α*33* transcripts was quantified using primer pairs: *V*α*19*F, 5'-GGTACAGTTACCTGCTTCTGAC-3', and *J*α*33*R, 5'-GATAGTTGCTATCCCTCACAGC-3' [52], and the results were normalized to TCRα constant region mRNA.

### Statistical analysis

Statistical analysis was performed with GraphPad Prism software (GraphPad, La Jolla, CA). When comparing experimental values from two groups of mice, one- or two-tailed student's t-tests were routinely used. When comparing experimental values from multiple groups, two-way ANOVA Bonferroni post-tests were used. Statistically significant differences are noted (***P < 0.001; **P < 0.01; *P < 0.05).

## Supporting Information

S1 Fig M3-restricted CD8^+^ T cells do not represent a significant population of Mtb-specific T cells in K^b-/-^D^b-/-^mice after Mtb infection. T cells from the lungs of K^b-/-^D^b-/-^ mice at day 30 post-infection were stimulated with unpulsed or CFP-pulsed K^b-/-^D^b-/-^ and K^b-/-^D^b-/-^M3^-/-^ BMDCs, respectively. The IFN-γ-secreting cells were quantified in an ELISPOT assay. Data shown are representative of two independent experiments, and are the mean ± SEM (n = 3 per experiment). ns, no statistical significance.
(TIF)

S2 Fig BMDCs from B6.C-H2^d^/bByJ mice lack Qa-2 expression but have other functional MHC Ib molecules. (A) The expression level of Qa-2 on BMDCs from C57BL/6, BALB/cJ and B6.C-H2^d^/bByJ (Qa-2^null^) mice were examined with an anti-Qa-2 antibody by flow cytometry. (B) The stimulatory capability of BMDCs from B6.C-H2d/bByJ (Qa-2^null^) and C57BL/6 mice to LemA-specific M3-restricted D7 T cells was comparable as shown in an IFN-γ ELISA.
(TIF)

S3 Fig γδ T cells do not expand significantly in the lung of K^b-/-^D^b-/-^M3^-/-^ mice during Mtb infection. Lymphocytes from the lung of K^b-/-^D^b-/-^M3^-/-^ and C57BL/6 mice before infection (naïve, n = 3) or at day 30 after Mtb infection (infected, n = 4) were analyzed by flow cytometry. Bar graphs depict the mean ± SEM of the percentage of γδ T cells in the lung of indicated mice. Data shown are representative of two independent experiments. ***P <0.001; ns, no statistical significance.
(TIF)

S4 Fig The expansion of CD4^-^CD8^-^ T cells in the lungs of Mtb-infected K^b-/-^D^b-/-^M3^-/-^ mice is β_2_m-independent. The percentages of CD4^-^CD8^-^ T cells (DN) in the lung of naïve or Mtb infected C57BL/6 (n = 4–6), K^b-/-^D^b-/-^M3^-/-^ (n = 4–6) and β_2_m^-/-^ (n = 3–6) mice at day 60 post-infection were analyzed by flow cytometry. Data shown are pooled from two independent experiments.
(TIF)

S5 Fig Mtb Antigens stimulate BMDCs to produce pro-inflammatory cytokines in part through the MyD88-dependent pathway. The supernatant from unpulsed, CFP-pulsed and PstS1-pulsed C57BL/6 and MyD88-/- BMDCs were harvested and subjected to cytometric beads assay for the detection of IL-12, IL-6 and IL-1β.
(TIF)

S6 Fig Increased bacterial burden in BALB/cByJ mice is associated with the lack of Qa-2-restricted CD8^+^ T cells. (A) BALB/cJ (n = 8) and BALB/cByJ mice (n = 7) were sacrificed on day 30 after low dose aerosol infection of Mtb H37Rv, lungs and spleens were harvested for plating to determine the bacterial burden. (B, C) T cells in the lungs of BALB/cJ (n = 4) and BALB/cByJ mice (n = 4) were stimulated for 18h with un-pulsed or CFP-pulsed BALB/cJ and BALB/cByJ BMDCs, respectively, and then harvested for intracellular staining of IFN-γ and TNF-α. The percentage of cytokine-producing CD8^+^ (B) and CD4^+^ (C) T cells were analyzed by flow cytometry. *P <0.05, **P <0.01, ns, no statistical significance.
(TIF)
