# Supplementary figures and images for: Nonclassical MHC Ib-restricted CD8+ T Cells Recognize Mycobacterium tuberculosis-Derived Protein Antigens and Contribute to Protection Against Infection
Source: PLoS Pathog. 2016 Jun 7;12(6):e1005688. doi: 10.1371/journal.ppat.1005688 (PMC4896622; doi:10.1371/journal.ppat.1005688)

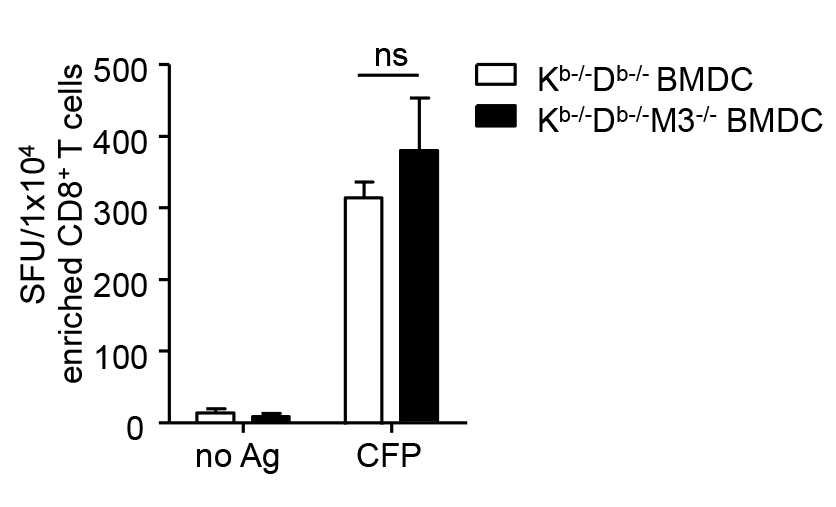

Supplement: S1 Fig — T cells from the lungs of Kb-/-Db-/- mice at day 30 post-infection were stimulated with unpulsed or CFP-pulsed Kb-/-Db-/- and Kb-/-Db-/-M3-/- BMDCs, respectively. The IFN-γ-secreting cells were quantified in an ELISPOT assay. Data shown are representative of two independent experiments, and are the mean ± SEM (n = 3 per experiment). ns, no statistical significance. (TIF) [file ppat.1005688.s001.tif]

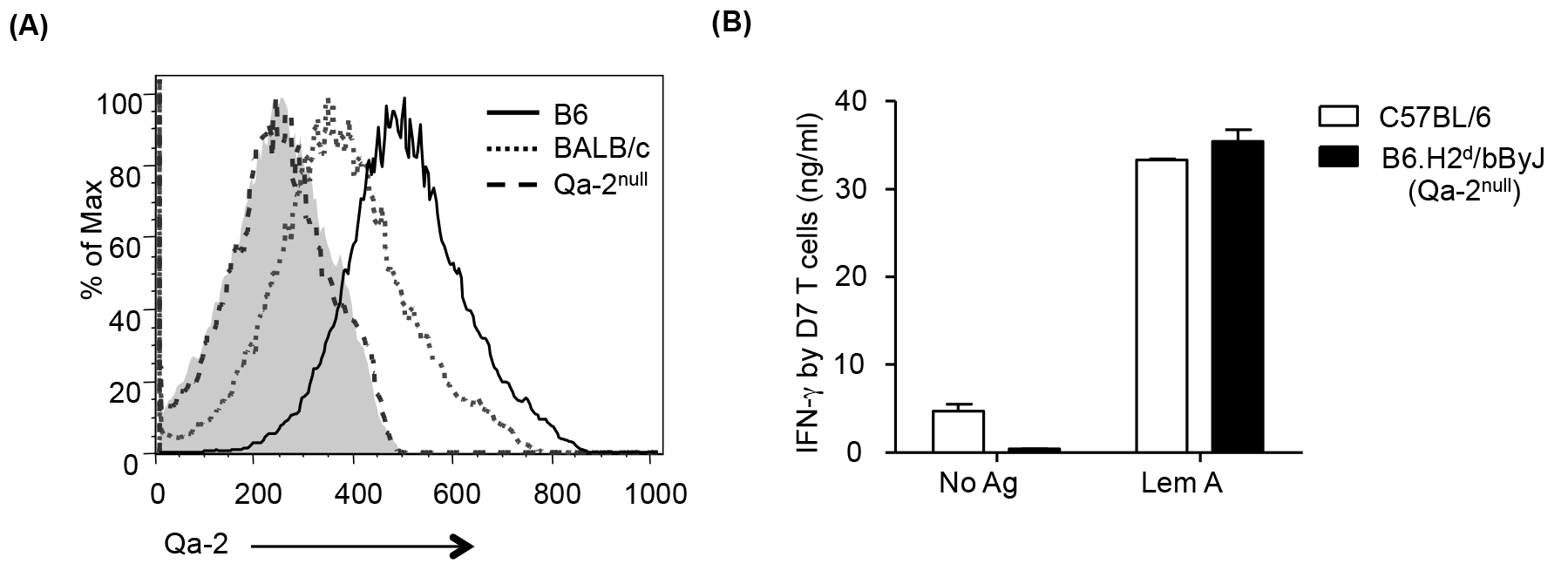

Supplement: S2 Fig — (A) The expression level of Qa-2 on BMDCs from C57BL/6, BALB/cJ and B6.C-H2d/bByJ (Qa-2null) mice were examined with an anti-Qa-2 antibody by flow cytometry. (B) The stimulatory capability of BMDCs from B6.C-H2d/bByJ (Qa-2null) and C57BL/6 mice to LemA-specific M3-restricted D7 T cells was comparable as shown in an IFN-γ ELISA. (TIF) [file ppat.1005688.s002.tif]

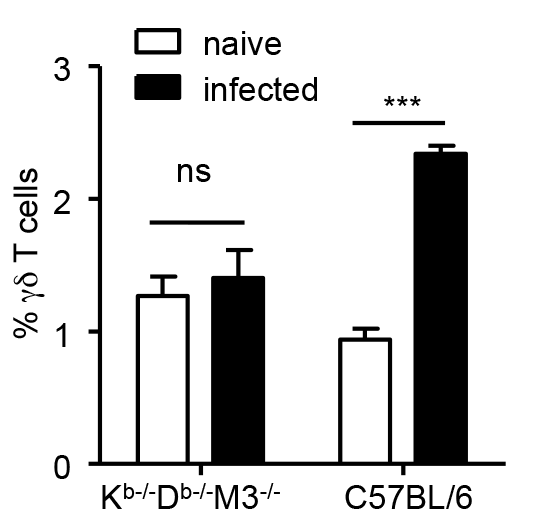

Supplement: S3 Fig — Lymphocytes from the lung of Kb-/-Db-/-M3-/- and C57BL/6 mice before infection (naïve, n = 3) or at day 30 after Mtb infection (infected, n = 4) were analyzed by flow cytometry. Bar graphs depict the mean ± SEM of the percentage of γδ T cells in the lung of indicated mice. Data shown are representative of two independent experiments. ***P <0.001; ns, no statistical significance. (TIF) [file ppat.1005688.s003.tif]

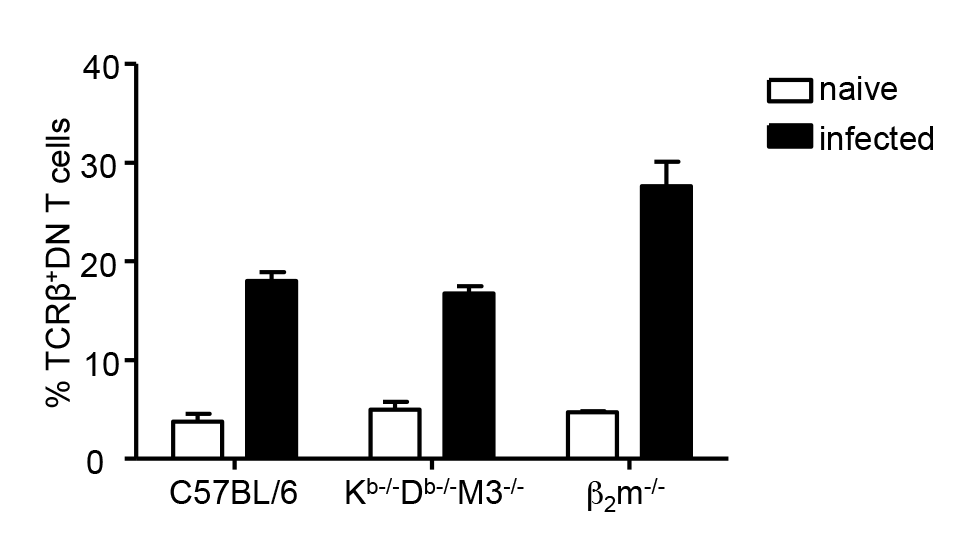

Supplement: S4 Fig — The percentages of CD4-CD8- T cells (DN) in the lung of naïve or Mtb infected C57BL/6 (n = 4–6), Kb-/-Db-/-M3-/- (n = 4–6) and β2m-/- (n = 3–6) mice at day 60 post-infection were analyzed by flow cytometry. Data shown are pooled from two independent experiments. (TIF) [file ppat.1005688.s004.tif]

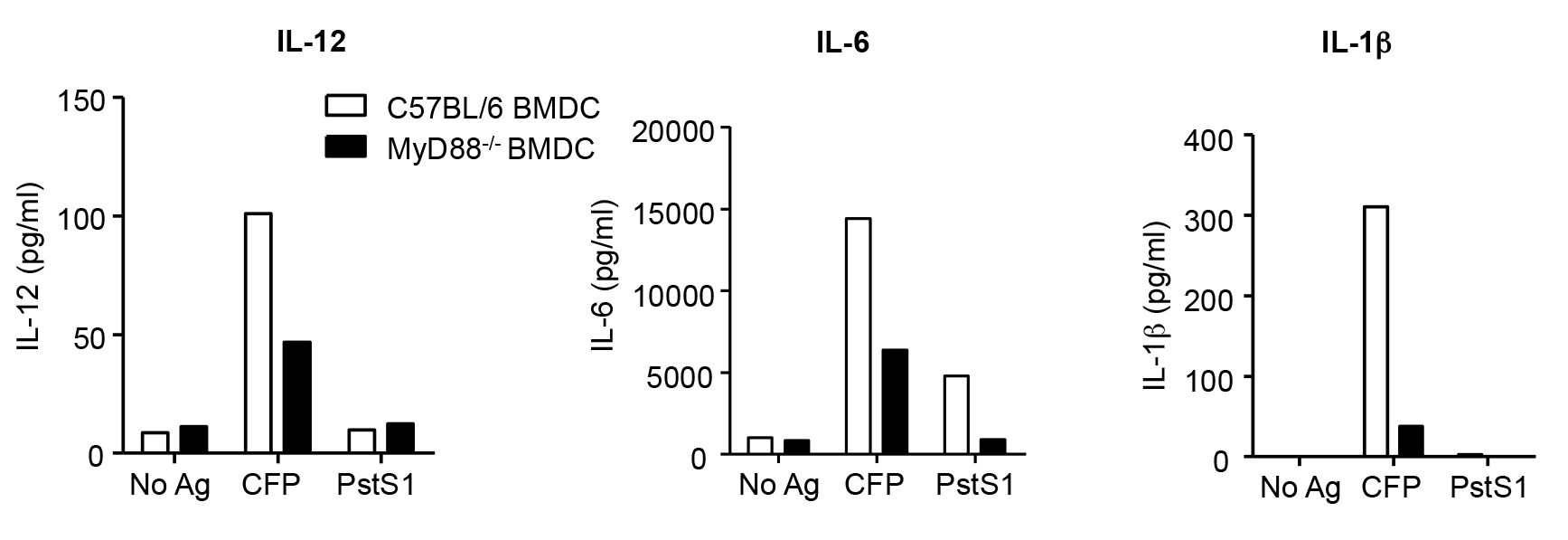

Supplement: S5 Fig — The supernatant from unpulsed, CFP-pulsed and PstS1-pulsed C57BL/6 and MyD88-/- BMDCs were harvested and subjected to cytometric beads assay for the detection of IL-12, IL-6 and IL-1β. (TIF) [file ppat.1005688.s005.tif]

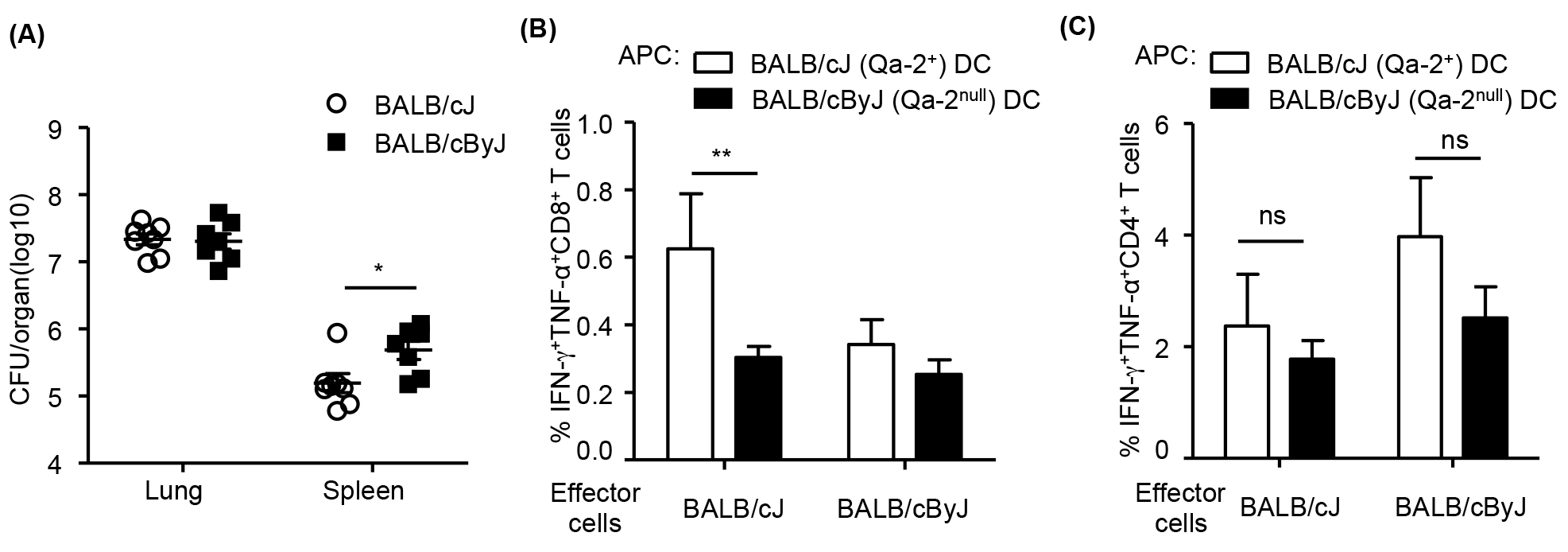

Supplement: S6 Fig — (A) BALB/cJ (n = 8) and BALB/cByJ mice (n = 7) were sacrificed on day 30 after low dose aerosol infection of Mtb H37Rv, lungs and spleens were harvested for plating to determine the bacterial burden. (B, C) T cells in the lungs of BALB/cJ (n = 4) and BALB/cByJ mice (n = 4) were stimulated for 18h with un-pulsed or CFP-pulsed BALB/cJ and BALB/cByJ BMDCs, respectively, and then harvested for intracellular staining of IFN-γ and TNF-α. The percentage of cytokine-producing CD8+ (B) and CD4+ (C) T cells were analyzed by flow cytometry. *P <0.05, **P <0.01, ns, no statistical significance. (TIF) [file ppat.1005688.s006.tif]
